# Supplementary material for: Enhancement of parvalbumin interneuron-mediated neurotransmission in the retrosplenial cortex of adolescent mice following third trimester-equivalent ethanol exposure
Source: Sci Rep. 2021 Jan 18;11:1716. doi: 10.1038/s41598-021-81173-z (PMC7814038; doi:10.1038/s41598-021-81173-z)
Supplement: Supplementary file 1 — Supplementary Information [file 41598_2021_81173_MOESM1_ESM.pdf]

Supplementary Materials for:

ENHANCEMENT OF PARVALBUMIN INTERNEURON-MEDIATED NEUROTRANSMISSION IN THE RETROSPLENIAL CORTEX OF ADOLESCENT MICE FOLLOWING THIRD TRIMESTER-EQUIVALENT ETHANOL EXPOSURE

Clark W. Bird, Glenna J. Chavez, Megan J. Barber, and C. Fernando Valenzuela

Department of Neurosciences, School of Medicine,
University of New Mexico Health Sciences Center
Albuquerque, New Mexico, USA

This PDF file includes:
Supplementary Figures 1 to 4
Supplemetary Tables 1 to 3

Supplemental Figure 1

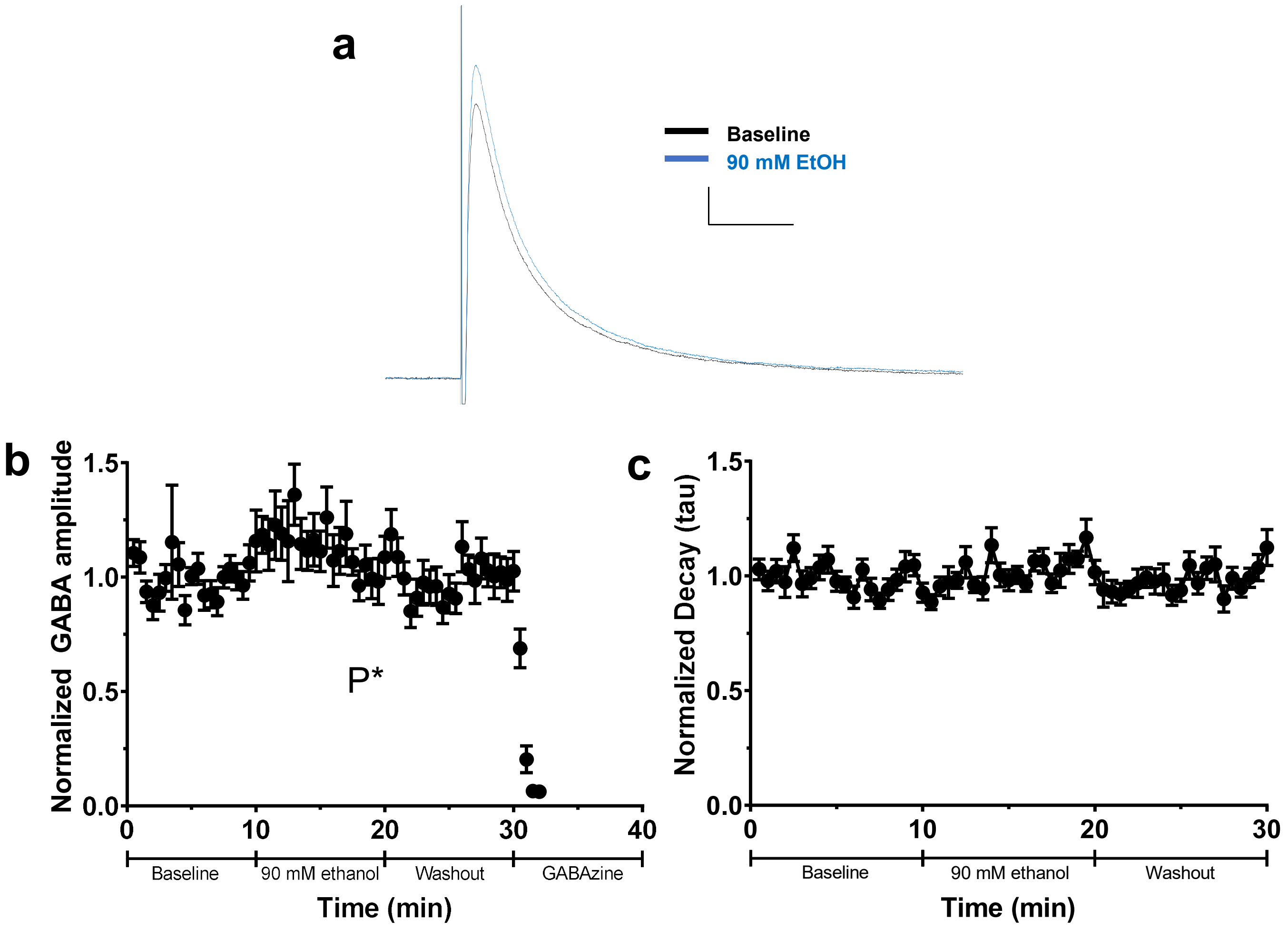

Supplemental Figure 1. Effect of acute ethanol application on evoked $GABA_A$ receptor-mediated postsynaptic current ($GABA_A$-ePSC) amplitude and decay in CA1 hippocampal pyramidal neurons. a) Average ePSC traces from CA1 pyramidal neurons during baseline (black trace) and the first 5 minutes of the acute 90 mM ethanol application phase. Scale bars = 40 ms, 20 pA. b) Normalized $GABA_A$-ePSC amplitudes during the baseline, 90 mM ethanol application, washout, and gabazine (25 μM) application phases. c) Normalized decay constants (tau) for CA1 pyramidal neurosn during the baseline, 90 mM ethanol application and washout phases. Data are presented as mean ± SEM. P* denotes a one-way repeated measures ANOVA p-value of 0.0294 for effect of phase comparing the last 5 min of baseline to the first 5 min of the 90 mM ethanol application phase and the first 5 min of the washout phase.

Supplemental Figure 2

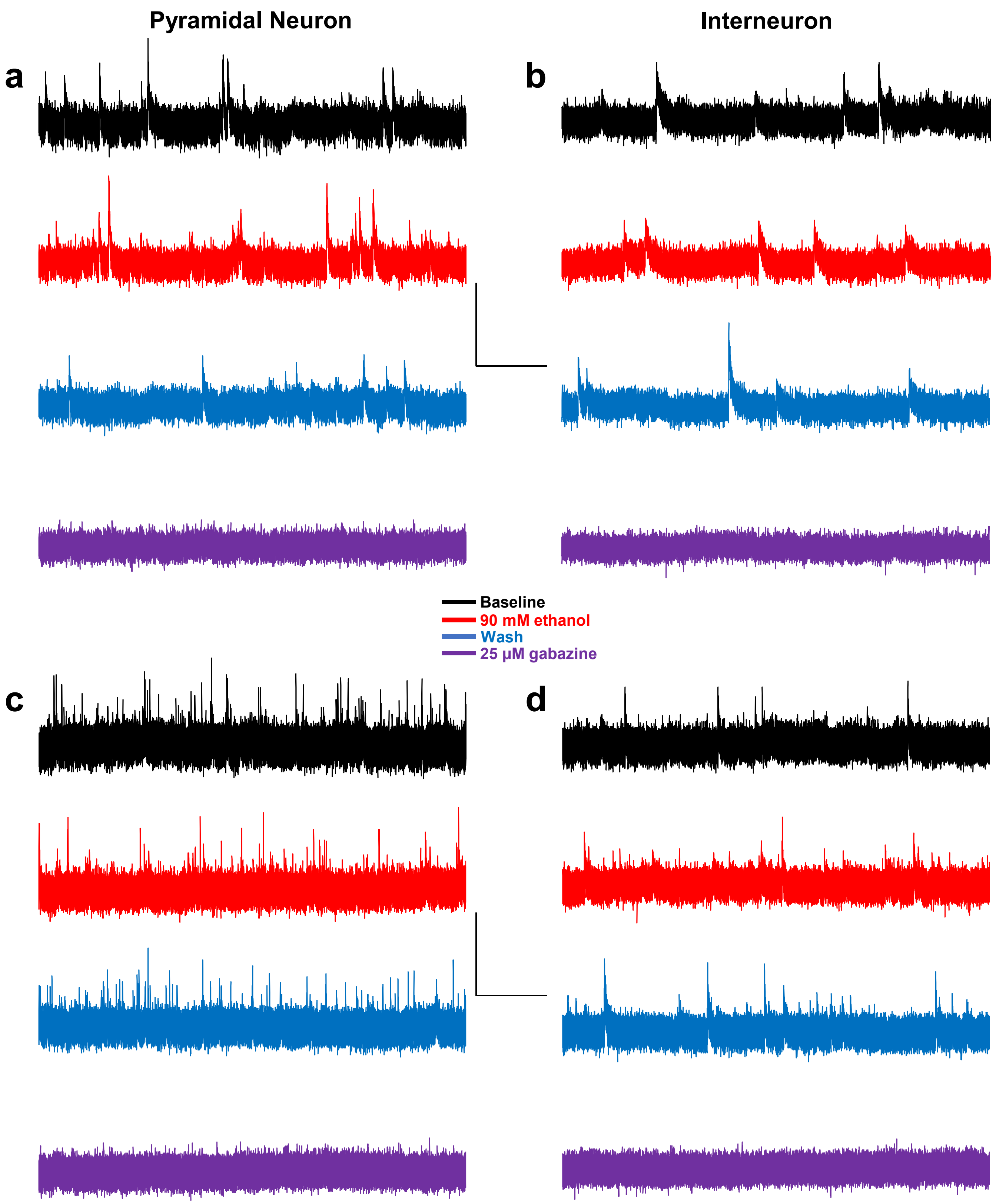

Supplemental Figure 2. Representative expanded and compressed sPSC traces from a pyramidal neuron and an interneuron demonstrating the effect of 90 mM ethanol application, washout, and 25 µM gabazine application. a) Expanded traces from a pyramidal neuron during baseline (black trace), 90 mM ethanol application (red trace), washout (blue trace), and 25 µM gabazine application (purple trace). b) expanded traces from an interneuron. Scale bar for expanded traces = 100 ms, 50 pA. c) compressed traces from a pyramidal neuron. d) compressed traces from an interneuron. Scale bar for compressed traces = 5 s, 50 pA.

Supplemental Figure 3

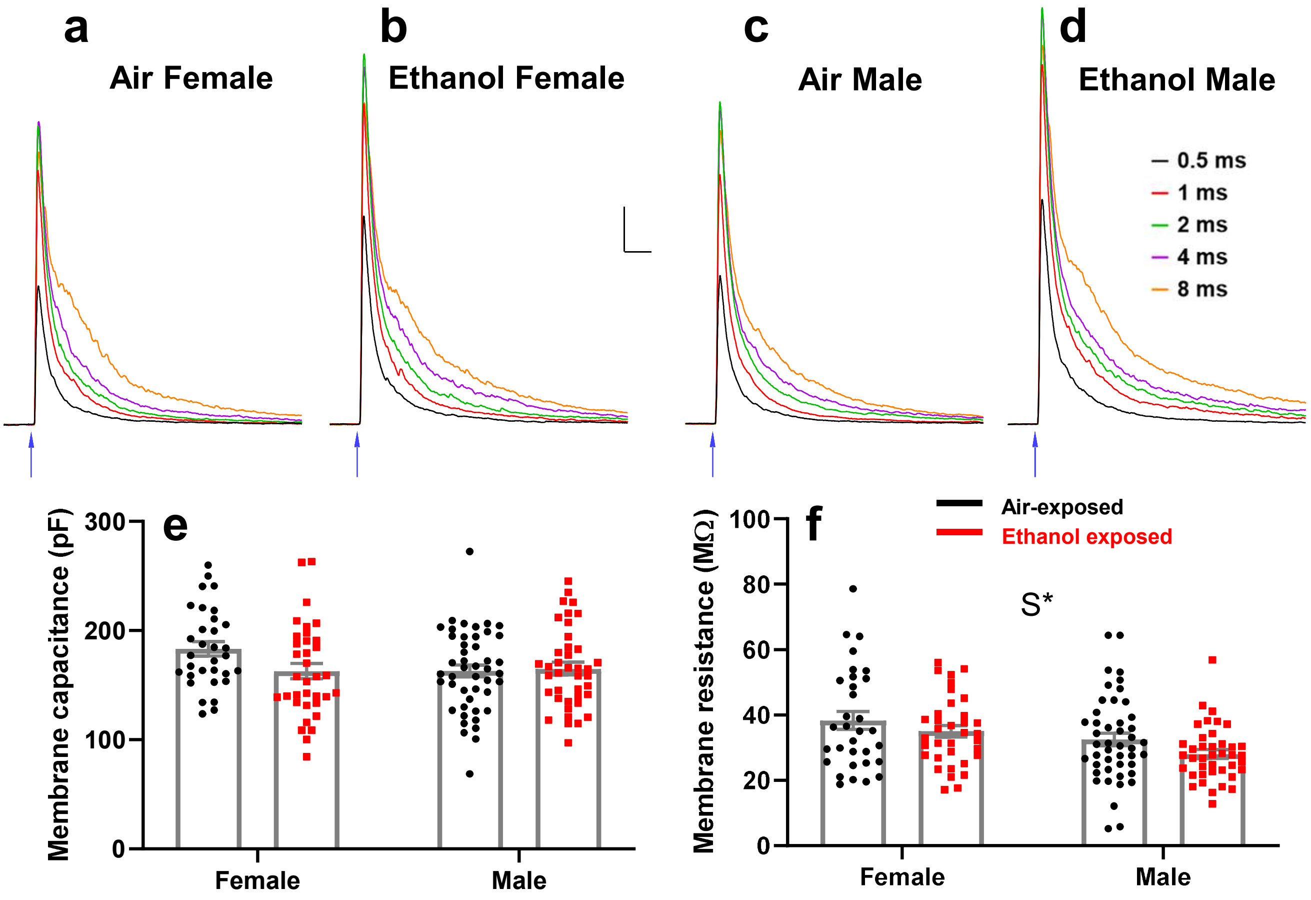

Supplemental Figure 3. Average optically-evoked inhibitory postsynaptic current (oIPSC) traces for each laser pulse duration in air- and ethanol-exposed animals and analysis of cell membrane capacitance and resistance presented separately by sex. a-d) Average oIPSC traces from air-exposed female (a), ethanol-exposed female (b), air-exposed male (c), and ethanol-exposed male (d) animals using 0.5 ms (black trace) 1 ms (red trace) 2 ms (green trace) 4 ms (purple trace) and 8 ms (orange trace) laser pulse durations. Scale bars = 20 ms,200 pA. Blue arrows indicate onset of laser pulse e) Membrane capacitances from recordings of optically-evoked inhibitory postsynaptic currents (oIPSC) presented separately by sex. Black circles are values from air-exposed animals, and red squares are values from ethanol-exposed animals. f) Membrane resistances from oIPSC recordings presented separately by sex. Asterisk (*) indicates a p-value of < 0.05. S indicates an effect of sex. Female air n = 32 cells from 8 animals from 7 litters, male air n = 46 cells from 9 animals from 8 litters; female ethanol n = 35 cells from 8 animals from 8 litters, male ethanol n = 40 cells from 8 animals from 7 litters. Data are presented as mean ± SEM.

Supplemental Figure 4

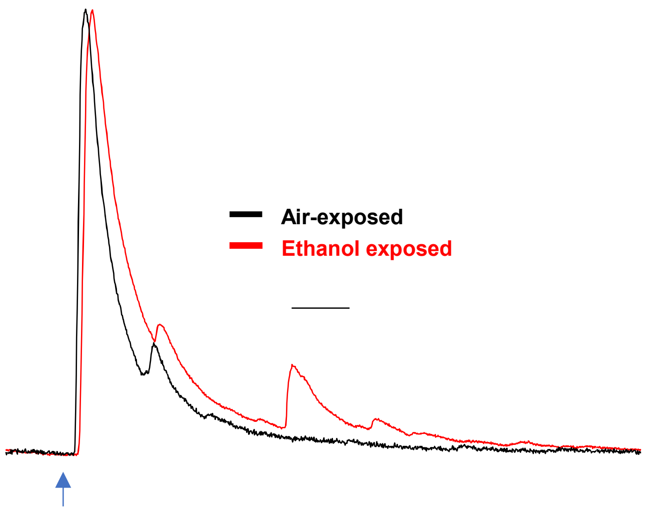

Supplemental Figure 4. Representative traces demonstrating asynchronous activity evoked by optogenetic stimulation. Traces show multiple oIPSC peaks evoked from 1 ms laser stimulation (blue arrow shows onset of 1 ms laser stimulation. The black trace is from an air-exposed control animal, the red trace is from an ethanol exposed animal. Traces are normalized by maximal peak amplitude. Scale bar = 10 ms.

Figure or results section	Experiment	Test	Measure	df	Test statistic	p value	Effect size measure	Effect size	Notes	Passes SW normality test?
Figure 2c	Effect of acute ethanol on ePSC amplitude	Repeated measures two-way ANOVA	Interaction: Exposure Phase X Cell Type	F(1.421,22.742)	0.369	0.6237	Partial eta squared	0.023	Did not pass Mauchly's test of sphericity, using Greenhouse-geisser corrected F-ratios and p-values	Yes: residuals pass
			Main effect: Exposure phase	F(1.421,22.742)	3.934	0.0467	Partial eta squared	0.197		
			Main effect: Cell type	F(1,16)	5.225	0.0362	Partial eta squared	0.246		
		Post-hoc multiple comparison	Baseline vs. 90 mM EtOH	t(17)	2.470	0.0732	Hedges' g	0.569	Bonferroni adjusted p-value	
			Baseline vs. Wash	t(17)	2.031	0.1746	Hedges' g	0.468	Bonferroni adjusted p-value	
			90 mM EtOH vs. Wash	t(17)	0.322	>0.9999	Hedges' g	0.074	Bonferroni adjusted p-value	
Figure 2d	Effect of acute ethanol on ePSC decay (tau)	Friedman's ANOVA	Main effect: Exposure phase	$\chi^2(2)$	2.111	0.3480	Kendall's W	0.059		No: residuals fail normality test
		Mann-Whitney U	Main effect: Cell type	U(n1 = 10, n2 = 8)	25	0.2031	r	0.314		
Figure 3c	Effect of acute flunitrazepam on ePSC amplitude	Repeated measures two-way ANOVA	Interaction: Exposure Phase X Cell Type	F(1.235,32.117)	0.376	0.5885	Partial eta squared	0.014	Did not pass Mauchly's test of sphericity, using Greenhouse-geisser corrected F-ratios and p-values	Yes: residuals pass
			Main effect: Exposure phase	F(1.235,32.117)	2.000	0.1648	Partial eta squared	0.071		
			Main effect: Cell Type	F(1,26)	11.562	0.0022	Partial eta squared	0.308		
Figure 3d	Effect of acute flunitrazepam on ePSC decay	Friedman's ANOVA	Main effect: Exposure phase	$\chi^2(2)$	39.071	<0.0001	Kendall's W	0.698		No: residuals fail normality test
		Mann-Whitney U	Main effect: Cell type	U(n1 = n2 = 14)	62	0.1035	r	0.313		
		Friedman's ANOVA post-hoc multiple comparisons (Dunn's multiple comparisons test)	Baseline vs. 1 μM flunitrazepam	U(n1 = n2 = 28)	z = 5.479	<0.0001	r	1.035	Bonferroni adjusted p-value	
			Baseline vs. Wash	U(n1 = n2 = 28)	z = 5.345	<0.0001	r	1.010	Bonferroni adjusted p-value	
			1 μM flunitrazepam vs. Wash	U(n1 = n2 = 28)	z = 0.134	>0.9999	r	0.025	Bonferroni adjusted p-value	
Figure 4c&d	Effect of acute ethanol on sPSC frequency	Repeated measures two-way ANOVA	Interaction: Exposure Phase X Cell Type	F(1.382, 17.969)	0.345	0.6344	Partial eta squared	0.026	Did not pass Mauchly's test of sphericity, using Greenhouse-geisser corrected F-ratios and p-values	Yes: residuals pass
			Main effect: Exposure phase	F(1.382, 17.969)	3.485	0.0670	Partial eta squared	0.211		
			Main effect: cell type	F(1,13)	9.980	0.0075	Partial eta squared	0.434		
Figure 4e&f	Effect of acute ethanol on sPSC amplitude	Repeated measures two-way ANOVA	Interaction: Exposure Phase X Cell Type	F(1.336, 17.371)	6.108	0.0173	Partial eta squared	0.320	Did not pass Mauchly's test of sphericity, using Greenhouse-geisser corrected F-ratios and p-values	Yes: residuals pass
			Main effect: Exposure phase	F(1.336, 17.371)	4.913	0.0314	Partial eta squared	0.274		
			Main effect: cell type	F(1,13)	0.137	0.7173	Partial eta squared	0.010		
		Post-hoc multiple comparison: within pyramidal neurons	Baseline vs. 90 mM EtOH	t(7)	1.381	0.6295	Hedges' g	0.274	Bonferroni adjusted p-value	
			Baseline vs. Wash	t(7)	1.373	0.6362	Hedges' g	0.192	Bonferroni adjusted p-value	
			90 mM EtOH vs. Wash	t(7)	1.029	>0.9999	Hedges' g	0.081	Bonferroni adjusted p-value	
		Post-hoc multiple comparison: within interneurons	Baseline vs. 90 mM EtOH	t(6)	1.110	0.9283	Hedges' g	0.216	Bonferroni adjusted p-value	
			Baseline vs. Wash	t(6)	2.357	0.1695	Hedges' g	0.856	Bonferroni adjusted p-value	
			90 mM EtOH vs. Wash	t(6)	2.234	0.2007	Hedges' g	0.642	Bonferroni adjusted p-value	
Figure 4g&h	Effect of acute ethanol on sPSC rise-time	Repeated measures two-way ANOVA	Interaction: Exposure Phase X Cell Type	F(2,26)	0.265	0.769524	Partial eta squared	0.020	Passed Mauchly's test of sphericity	Yes: residuals pass
			Main effect: Exposure phase	F(2,26)	2.763	0.081654	Partial eta squared	0.175		
			Main effect: cell type	F(1,13)	1.448	0.250276	Partial eta squared	0.100		
Figure 4i&j	Effect of acute ethanol on sPSC decay (tau)	Repeated measures two-way ANOVA	Interaction: Exposure Phase X Cell Type	F(2,24)	0.123	0.885017	Partial eta squared	0.010	Passed Mauchly's test of sphericity; Removed one interneuron with outlier wash decay value	Yes: residuals pass
			Main effect: Exposure phase	F(2,24)	0.834	0.446609	Partial eta squared	0.065		
			Main effect: cell type	F(1,12)	13.543	0.003148	Partial eta squared	0.530		
Figure 5e	% Colocalization PV-tdTomato/ChR2	Mann-Whitney U	Effect of P7 ethanol exposure	U(n1 = n2 = 5)	12	>0.9999	r	0.047		No
Figure 5f	% Nonspecific transgene expresion	Unpaired t-test	Effect of P7 ethanol exposure	t(8)	0.406	0.6954	Hedges' g	0.232		Yes
Supplemental figure 1b	Effect of acute ethanol on ePSC amplitude (CA1 pyramidal neurons)	One-way ANOVA	Main effect: Exposure phase	F(2,24)	4.101	0.0294	Partial eta squared	0.255	Passed Mauchly's test of sphericity. Baseline is compared to first 5 minutes of EtOH and wash phases. Bonferroni corrected p-values for muliple comparisons	Yes: residuals pass
		Post-hoc multiple comparison	Baseline vs. 90 mM EtOH	t(12)	2.258	0.1299	Hedges' g	0.396		
			Baseline vs. Wash	t(12)	0.663	>0.9999	Hedges' g	0.106		
			90 mM EtOH vs. Wash	t(12)	2.405	0.0996	Hedges' g	0.481		
Supplemental figure 1c	Effect of acute ethanol on ePSC decay (tau, CA1 pyramidal neurons)	One-way ANOVA	Main effect: Exposure phase	F(2,24)	0.484	0.6224	Partial eta squared	0.039	Passed sphericity test. Baseline compared to first 5 minutes of EtOH and wash phases	Yes: residuals pass

Supplemental Table 1: Comprehensive collection of statistical analyses for Experiment 1 and IHC analyses from Experiment 2.
Detailed information regarding specific tests used, measures examined, degrees of freedom, test statistics, p-values, effect sizes, and results of normality testing are presented.

Figure	Experiment	Does random effect of litter significantly improve LMM?	Do heterogenous error variances significantly improve LMM?	Exposure	Sex	Laser	Exposure*Sex	Exposure*Laser	Sex*Laser	Exposure*Sex*Laser	Mann-Whitney U: Exposure	Mann-Whitney U: Sex	Notes
Supplemental Figure 3e	Effect of P7 ethanol exposure on oIPSC cell capacitance	No	No	$F(1,149) = 2.137$<br>$p = 0.1459$<br>$g = 0.186$	$F(1,149) = 2.089$<br>$p = 0.1505$<br>$g = 0.221$	-	$F(1,149) = 3.274$<br>$p = 0.0724$	-	-	-	-	-	
Supplemental figure 3f	Effect of P7 ethanol exposure on oIPSC membrane resistance	Yes	Yes	$F(1,19.644) = 0.763$<br>$p = 0.3930$<br>$g = 0.288$	$F(1,95.267) = 4.199$<br>$p = 0.0432$<br>$g = 0.498$	-	$F(1,95.267) = 0.004$<br>$p = 0.9471$	-	-	-	$U(n1 = 78\ n2 = 75) = 2550$<br>$p = 0.1711$<br>$r = 0.111$	$U(n1 = 67, n2 = 86) = 2094$<br>$p = 0.0038$<br>$r = 0.234$	
Figure 6c	Effect of P7 ethanol exposure on oIPSC amplitude	No	Yes	$F(1,146.828) = 5.009$<br>$p = 0.0267$<br>$g = 0.328$	$F(1,146.828) = 0.518$<br>$p = 0.4730$<br>$g = 0.075$	$F(4,138.866) = 73.641$<br>$p < 0.0001$	$F(1,146.828) = 0.082$<br>$p = 0.7745$	$F(4,138.866) = 1.012$<br>$p = 0.4035$	$F(1,138.866) = 1.015$<br>$p = 0.4019$	$F(4,138.866) = 0.712$<br>$p = 0.5850$	$U(n1 = 387,n2 = 372) = 57494$<br>$p < 0.0001$<br>$r = 0.174$	$U(n1 = 333, n2 = 426) = 67014$<br>$p = 0.1915$<br>$r = 0.047$	
Figure 6d	Effect of P7 ethanol exposure on oIPSC current density	Yes	No	$F(1,40.564) = 4.044$<br>$p = 0.051$<br>$g = 0.384$	$F(1,108.138) = 0.687$<br>$p = 0.4089$<br>$g = 0.157$	$F(4,38.243) = 66.409$<br>$p < 0.0001$	$F(1,108.138) = 0.202$<br>$p = 0.6542$	$F(4,38.243) = 1.283$<br>$p = 0.2936$	$F(4,96.088) = 0.816$<br>$p = 0.5179$	$F(4,96.088) = 0.923$<br>$p = 0.4538$	$U(n1 = 386, n2 = 372) = 54471$<br>$p < 0.0001$<br>$r = 0.209$	$U(n1 = 332, n2 = 426) = 64027$<br>$p = 0.0253$<br>$r = 0.081$	
Figure 6e	Effect of P7 ethanol exposure on oIPSC charge	Yes	Yes	$F(1,20.848) = 5.907$<br>$p = 0.0242$<br>$g = 0.424$	$F(1,62.016) = 0.848$<br>$p = 0.3606$<br>$g = 0.123$	$F(4,88.351) = 133.734$<br>$p < 0.0001$	$F(1,62.016) = 0.682$<br>$p = 0.4121$	$F(4,88.351) = 2.849$<br>$p = 0.0284$	$F(4,292.749) = 0.682$<br>$p = 0.6047$	$F(4,292.749) = 1.401$<br>$p = 0.2338$	$U(n1 = 381, n2 = 368) = 52361$<br>$p < 0.0001$<br>$r = 0.219$	$U(n1 = 328, n2 = 421) = 63326$<br>$p = .0516$<br>$r = 0.071$	Compound symmetry covariance matrix for repeated measure residuals
Figure 6f	Effect of P7 ethanol exposure on oIPSC half-width	Yes	Yes	$F(1,26.478) = 2.315$<br>$p = 0.1400$<br>$g = 0.270$	$F(1,89.647)$<br>$p = 0.3165$<br>$g = 0.091$	$F(4,40.164) = 51.786$<br>$p < 0.0001$	$F(1,89.647) = 0.736$<br>$p = 0.3932$	$F(4,40.164) = 0.604$<br>$p = 0.6619$	$F(4,84.740) = 1.136$<br>$p = 0.3451$	$F(4,84.740) = 0.496$<br>$p = 0.7389$	$U(n1 = 383, n2 = 370) = 60535$<br>$p = 0.0005$<br>$r = 0.126$	$U(n1 = 331, n2 = 422) = 64263$<br>$p = .0597$<br>r = 0.069	
Figure 6g	Effect of P7 ethanol exposure on oIPSC rise time	No	Yes	$F(1,110.583) = 11.906$<br>$p = 0.0008$<br>$g = 0.359$	$F(1,110.583) = 0.397$<br>$p = 0.5297$<br>$g = 0.119$	$F(4,102.143) = 15.353$<br>$p < 0.0001$	$F(1,110.583) = 0.713$<br>$p = 0.4001$	$F(4,102.143) = 4.142$<br>$p = 0.0038$	$F(4,102.143) = 1.811$<br>$p = 0.1324$	$F(4,102.143) = 0.567$<br>$p = 0.6870$	$U(n1 = 349, n2 = 324) = 47028$<br>$p = 0.0002$<br>$r = 0.145$	$U(n1 = 288, n2 = 385) = 48535$<br>$p = 0.0057$<br>$r = 0.107$	
Figure 6h	Effect of P7 ethanol exposure on oIPSC asynchronous activity	No	Yes	$F(1,137.439) = 13.368$<br>$p = 0.0004$<br>$g = 0.387$	$F(1,137.439) = 0.706$<br>$p = 0.4020$<br>$g = 0.066$	$F(4,133.965) = 126.460$<br>$p < 0.0001$	$F(1,137.439) = 1.804$<br>$p = 0.1815$	$F(4,133.965) = 3.519$<br>$p = 0.0091$	$F(4,133.965) = 0.755$<br>$p = 0.5564$	$F(4,133.965) = 0.563$<br>$p = 0.6901$	$U(n1 = 373, n2 = 371) = 54031.5$<br>$p < 0.0001$<br>$r = 0.190$	$U(n1 = 324, n2 = 420) = 64434.5$<br>$p = 0.2135$<br>$r = 0.046$	
Figure 7b	Effect of P7 ethanol exposure on oIPSC PPR amplitude	No	No	$F(1,79) = 0.377$<br>$p = 0.5408$<br>$g = 0.159$	$F(1,79) = 5.119$<br>$p = 0.0264$<br>$g = 0.507$	-	$F(1,79) = 0.169$<br>$p = 0.6821$	-	-		-	-	
Figure 7c	Effect of P7 ethanol exposure on oIPSC PPR total charge	No	No	$F(1,79) = 1.521$<br>$p = 0.2211$<br>$g = 0.291$	$F(1,79) = 5.667$<br>$p = 0.0338$<br>$g = 0.489$	-	$F(1,79) = 0.182$<br>$p = 0.6709$	-	-		-	-	

Supplemental Table 2: Results from linear mixed-model analyses performed in Experiment 2.

Details from LMM model building are presented, indicating if including random effect of litter or heterogeneous error variances for vapor chamber exposure conditions significantly improved LMM. F-ratios and p-values are presented for vapor chamber exposure effects, sex effects, repeated measure laser pulse duration effects, and two- and three-way interactions between these effects. Hedges' g effect sizes are presented for exposure and sex effects. Non-Parametric Mann-Whitney U tests are presented (including effect size as r) for exposure and sex effects from any LMM with residuals that did not pass ($p > 0.05$) a Shapiro-Wilkes normality test.

Figure or results section	Experiment	Test statistic and degrees of freedom	p-value	Effect size (Hedges' *g*)	Mann-Whitney U: exposure	Notes
Figure 6e	Effect of P7 ethanol exposure on oIPSC charge: 0.5 msec laser pulse	t(35) = 0.396	> 0.99	0.680	U(n1 = 73, n2 = 73) = 1793, p = 0.0032, r = 0.282	Bonferroni corrected p-value
	Effect of P7 ethanol exposure on oIPSC charge: 1 msec laser pulse	t(34) = 1.922	0.3151	0.595	U(n1 = 77, n2 = 74) = 1947, p = 0.0056, r = 0.265	Bonferroni corrected p-value
	Effect of P7 ethanol exposure on oIPSC charge: 2 msec laser pulse	t(34) = 1.981	0.2782	0.455	U(n1 =77, n2 = 74) = 2091, p = 0.0239, r = 0.230	Bonferroni corrected p-value
	Effect of P7 ethanol exposure on oIPSC charge: 4 msec laser pulse	t(34) = 2.526	0.0814	0.548	U(n1 = 76, n2 = 73) = 1882, p = 0.0035, r = 0.277	Bonferroni corrected p-value
	Effect of P7 ethanol exposure on oIPSC charge: 8 msec laser pulse	t(34) = 3.363	0.0096	0.493	U(n1 = 78, n2 = 74) = 2069, p = 0.0130, r = 0.244	Bonferroni corrected p-value
Figure 6g	Effect of P7 ethanol exposure on oIPSC rise time: 0.5 msec laser pulse	t(107) = 3.472	0.0037	0.570	U(n1 = 75, n2 = 66) = 1926, p = 0.1165, r = 0.191	Bonferroni corrected p-value
	Effect of P7 ethanol exposure on oIPSC rise time: 1 msec laser pulse	t(105) = 3.783	0.0013	0.540	U(n1 = 72, n2 = 68) = 1881, p = 0.0904, r = 0.200	Bonferroni corrected p-value
	Effect of P7 ethanol exposure on oIPSC rise time: 2 msec laser pulse	t(124) = 0.946	> 0.99	0.215	U(n1 = 70, n2 = 65) = 1988, p > 0.99, r = 0.109	Bonferroni corrected p-value
	Effect of P7 ethanol exposure on oIPSC rise time: 4 msec laser pulse	t(110) = 2.635	0.0482	0.390	U(n1 = 67, n2 = 63) = 1730, p = 0.3815, r = 0.156	Bonferroni corrected p-value
	Effect of P7 ethanol exposure on oIPSC rise time: 8 msec laser pulse	t(110) = 2.635	0.0656	0.351	U(n1 = 65, n2= 62) = 1752, p > 0.99, r = 0.113	Bonferroni corrected p-value
Figure 6h	Effect of P7 ethanol exposure on oIPSC asynchronous activity: 0.5 msec laser pulse	t(109) = 3.381	0.0050	0.565	U(n1 = 72, n2 = 71) = 1819.5 p = 0.0120, r = 0.254	Bonferroni corrected p-value
	Effect of P7 ethanol exposure on oIPSC asynchronous activity: 1 msec laser pulse	t(113) = 4.322	0.0002	0.819	U(n1 = 71, n2 = 75) = 1659.5 p = 0.0004, r = 0.328	Bonferroni corrected p-value
	Effect of P7 ethanol exposure on oIPSC asynchronous activity: 2 msec laser pulse	t(123) = 3.620	0.0021	0.663	U(n1 = 74, n2 = 75) = 1921 p = 0.0057, r = 0.266	Bonferroni corrected p-value
	Effect of P7 ethanol exposure on oIPSC asynchronous activity: 4 msec laser pulse	t(144) = 3.191	0.0087	0.532	U(n1 = 78, n2 = 75) = 2096 p = 0.0122, r = 0.245	Bonferroni corrected p-value
	Effect of P7 ethanol exposure on oIPSC asynchronous activity: 8 msec laser pulse	t(149) = 1.922	0.2824	0.337	(U(n1 = 78, n2 = 75) = 2311 p = 0.1247, r = 0.181	Bonferroni corrected p-value

Supplemental Table 3: Post hoc examination of exposure by laser interactions with a p-value of < 0.05 from LMM analyses.
Non-parametric Mann-Whitney U tests for exposure effects within each laser pulse duration are presented for the effect of ethanol exposure on oIPSC total charge, oIPSC rise time, and oIPSC asynchronous activity. Data presented include: results of parametric post-hoc tests (not discussed in text as data violated normality assumptions), Mann-Whitney U test statistics with group sample sizes, p-values, and effect sizes as *r*. All p-values are Bonferroni corrected for the number of multiple comparisons made.
